# South Pacific influence on the termination of El Niño in 2014

**DOI:** 10.1038/srep30341

**Published:** 2016-07-28

**Authors:** Yukiko Imada, Hiroaki Tatebe, Masahiro Watanabe, Masayoshi Ishii, Masahide Kimoto

**Affiliations:** 1 Meteorological Research Institute, Japan Meteorological Agency, 1-1 Nagamine, Tsukuba, Ibaraki 305-0052, Japan; 2 Japan Agency for Marine-Earth Science and Technology, 3173-25 Showa-machi, Kanazawa-ku, Yokohama, Kanagawa 236-0001, Japan; 3 Atmosphere and Ocean Research Institute, the University of Tokyo, 5-1-5 Kashiwanoha, Kashiwa, Chiba 277-8568, Japan

## Abstract

The El Niño/Southern Oscillation (ENSO) is the dominant mode of climate variability affecting worldwide extreme weather events; therefore, improving ENSO prediction is an important issue. In this regard, a peculiar time evolution of ENSO in 2014 posed a challenge to the climate science community. Despite the observance of several precursors for a strong El Niño to develop during the summer and autumn, cold sea surface temperature (SST) anomalies appeared unexpectedly to the south of the equatorial cold tongue, which prevented development of an El Niño event in the late summer. Several hypotheses have been raised to explain the unmaterialized El Niño in 2014, but complete understanding of processes responsible for terminating this event has not yet been obtained. Here we show, using observations and extended seasonal prediction experiments with a climate model, that cold off-equatorial subsurface water in the South Pacific Ocean penetrated into the equatorial region along the slanted isopycnal surface via the mean advection, and it prevented the El Niño evolution in 2014. The negative subsurface temperature anomalies in the off-equatorial South Pacific Ocean were persistent throughout the last decade, and additional numerical simulations indicated that they contributed to the suppression of El Niño events during the 2000s.

In the first half of 2014, eastward propagation of large downwelling Kelvin waves was observed from February to June 2014, triggered by a series of westerly wind events (WWEs)^1^. The signal was visible in longitude-time plots of anomalies of vertically averaged temperature for the upper 300 m (VAT300) along the equator (Fig. S1b). The amplitude of the Kelvin waves was comparable to those observed during 1997, which brought the largest ever El Niño event in the winter of 1997–1998^2^. Thus, most operational forecasting models predicted the development of a large El Niño event in the following summer and autumn^3^. In 2014, however, the downwelling Kelvin waves disappeared by July, and SST did not increase in the eastern equatorial Pacific (Fig. S1a,b). Finally, negative SST anomalies spread over the cold tongue region, and warm SST anomalies remained only in the off-equatorial North Pacific Ocean in August 2014 (Fig. 1a). Several hypotheses have been raised to explain the unmaterialized El Niño in 2014, but complete understanding of processes responsible for terminating this El Niño have not yet been obtained. Although a few recent papers focused on the influence of the absence of sustained westerly wind anomalies in mid-2014^1^,^2^,^4^, no studies examined the influence of the off-equatorial subsurface anomalies in the South Pacific Ocean.

In this study, we investigated the role of the South Pacific subsurface anomalies in this event based on a one-year prediction of our climate model initialized on 1 November 2013 that reproduced the termination of El Niño in 2014 and an additional sensitivity experiment in which the penetration of South Pacific anomalies into the equatorial region was blocked. Furthermore, using long-term observations and extended model simulations, we investigated the origin of the South Pacific subsurface anomalies.

## Results

### Prediction of ENSO 2014 by MIROC5

**Figure 1:**
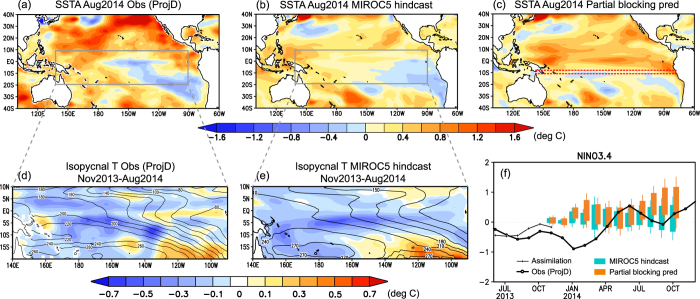
Observed and simulated anomalies in the Pacific Ocean in 2014. (**a**–**c**) SST anomalies for the observation (**a**), the hindcast (**b**), and the partial-blocking prediction (**c**) in August 2014. The red dashed box in (**c**) indicates the blocked area. (**d**,**e**) Ocean temperature anomalies (shading) and depth (contour, m) of the 25–26 kg m^−3^ isopycnal layer averaged from November 2013 to August 2014 for the observation (**d**) and the hindcast (**e**). (**f**) Nino3.4 indices for the observation (black), the hindcast (light blue), and the partial-blocking prediction (orange). Bars show the range from minimum to maximum values of the ensemble, and boxes show the range of the ensemble spread. GrADS version 1.9b4 (Free Software - http://cola.gmu.edu/grads/gadoc/COPYRIGHT) was used for this figure.

Interestingly, the seasonal prediction system^5^ based on a coupled atmosphere–ocean general circulation model (AOGCM) called the Model for Interdisciplinary Research on Climate version 5 (MIROC5)^6^, initialized on 1 November 2013 (i.e., lead time of nine months), predicted negative SST anomalies in the cold tongue region and warm SST anomalies in the off-equatorial North Pacific Ocean in August 2014 (Fig. 1b). We examined a common feature in the Pacific Ocean between the observation and the MIROC5 forecast and found a noteworthy aspect in the lower part of thermocline, which is represented as the isopycnal layer between 25 and 26 kg m^−3^ (reference depths are shown in Fig. S2a,b). Near the equator, the isopycnal temperature anomalies were below normal throughout the year (Fig. S1c). The horizontal distribution of isopycnal temperature anomalies shown in Fig. 1d indicates that those negative anomalies near the equator were connected to anomalies in the western off-equatorial South Pacific Ocean. It is known that there is a shallow meridional overturning circulation, a so-called subtropical cell (STC), that involves subtropical subduction, equatorward flows in the pycnocline, equatorial upwelling, and poleward surface Ekman flows^7^,^8^. In the South Pacific Ocean, the flow travels northwestward from the western off-equatorial South Pacific to the eastern and central equatorial Pacific along the thermocline (an example of streamlines is shown in Fig. S2c). There is a hypothesis that temperature anomalies in the subtropics are advected by the mean circulation of STCs into the tropics and impact tropical SST variability on decadal and interdecadal time scales (i.e. 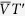 mechanism)^9^. This implies that the El Niño development in 2014 might have been interrupted by a deep oceanic slow process. The MIROC5 prediction also represented negative temperature anomalies from the western off-equatorial South Pacific to the western and central equatorial Pacific throughout the year (Fig. 1e), as was seen in the observation data (Fig. 1d). Note that there is no significant coupling between the off-equatorial north Pacific Ocean and the equatorial region via advection of temperature anomalies along the thermocline^10^,^11^.

### Partial-blocking prediction

**Figure 2:**
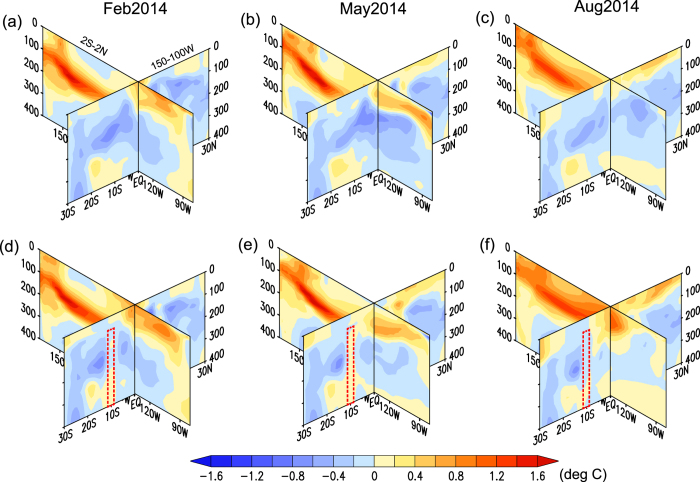
Meridional and zonal distribution of simulated subsurface temperature anomalies in the tropical Pacific Ocean. Latitude-depth (averaged between 150°W and 90°W) and longitude-depth (along the equator) plots of predicted ocean temperature anomalies in February (**a**,**d**), May (**b**,**e**) and August (**c**,**f**) 2014. (**a**–**c)** Show the MIROC hindcast and (**d**–**f**) show the partial-blocking prediction; both were initialized on 1 November 2013. The red dashed lines indicate columns where the temperature and salinity were restored to the model climatological values in the partial-blocking experiment.

To examine whether deep oceanic processes in the off-equatorial South Pacific Ocean were crucial to the interruption of the El Niño development in 2014, we conducted another set of prediction experiments, in which penetration of anomalies from the off-equatorial South Pacific Ocean to the equatorial strip was blocked by restoring temperature and salinity around 10°S to the monthly climatological values (hereafter referred to as the partial-blocking prediction, see Methods). When the penetration of South Pacific anomalies into the equatorial region was blocked, warming in the eastern equatorial Pacific occurred in August 2014 (Fig. 1c) unlike observations and the original prediction (Fig. 1ab). Figure 1f shows the Nino3.4 indices (SST anomalies averaged between 170°–150°W, 5°S–5°N) for the observation data, the original prediction, and the partial-blocking prediction. It illustrates that an El Niño would have developed steadily from summer to autumn of 2014 if there had not been an influence from the South Pacific Ocean. Figure 2 shows the meridional (averaged between 150°W–90°W) and equatorial sections of ocean temperature anomalies from the original prediction and the partial-blocking prediction that were initialized on 1 November 2013. At the beginning of the prediction, there were negative temperature anomalies in the subsurface South Pacific Ocean between 15°S and 10°S (Fig. 2a,d). As time progressed, the negative anomalies penetrated equatorward and interrupted development of El Niño in the original prediction (Fig.2b,c). On the other hand, the negative anomalies in the South Pacific Ocean could not impact the equatorial processes, and warm temperature anomalies developed in association with the downwelling Kelvin waves in the partial-blocking experiment (Fig. 2e,f).

**Figure 3:**
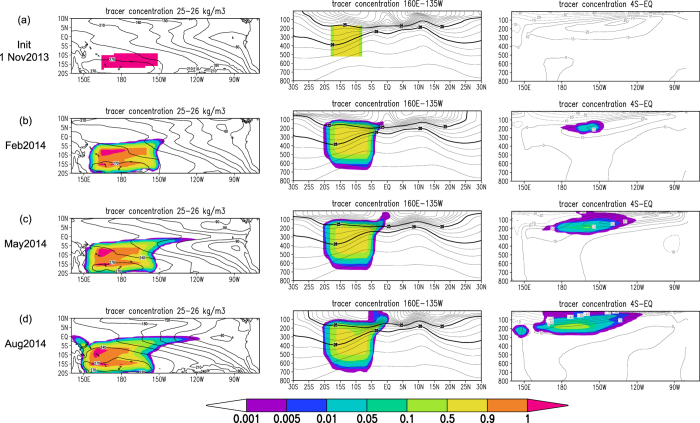
Penetration of subsurface water parcels from the South Pacific. **(a–d)** Passive tracer concentrations (shading) at the initial date (1 November 2013; **a**), February (**b**), May (**c**), and August 2014 (**d**) at the 25–26 kg m^−3^ isopycnal surface (left), in the 160°E–135°W meridional section (middle), and in the 4°S-equator zonal section (right). The contours in the left column indicate mean depth (m) of the 25–26 kg m^−3^ pycnocline. The contours in the middle column indicate mean density with 0.25 kg m^−3^ intervals; 25 and 26 kg m^−3^ are highlighted with thick curves. The contours in the right column indicate mean zonal velocity (m s^−1^). GrADS version 1.9b4 (Free Software - http://cola.gmu.edu/grads/gadoc/COPYRIGHT) was used for this figure.

To examine the propagation path and time scale of the temperature anomalies, we also conducted a tracer experiment in which passive tracers were put in the subsurface western off-equatorial South Pacific Ocean on 1 November 2013 (Fig. 3a). We confirmed that the tracers were advected by the ocean mean current and approached the surface in the central to eastern equatorial Pacific in August 2014 (Fig. 3b–d). Once the tracers passed through the equatorial strip, they were transported by the Equatorial Under Current (EUC) across the equatorial Pacific within a year. This fast displacement of water parcels over nine months can occur given an approximate zonal velocity of 10^−3^ km s^−1^ for the EUC and an approximate zonal distance of 1.4 × 10^4^ km for the equatorial Pacific (Fig. 3, right column).

### Origin of the South Pacific subsurface anomalies

**Figure 4:**
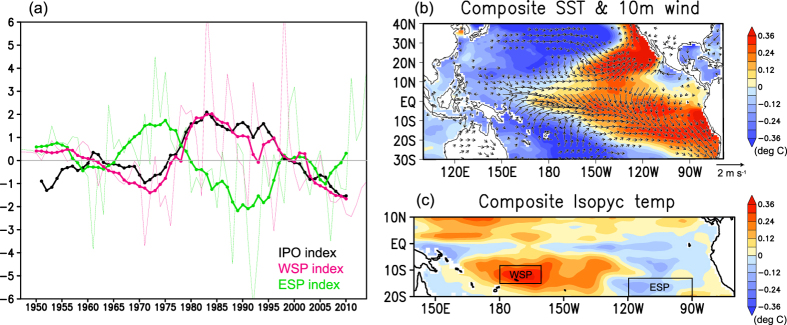
Observed IPO and related subsurface temperature variability. (**a**) Normalized time series of the IPO index (black), the isopycnal temperature in the western South Pacific Ocean (WSP, magenta), and the eastern South Pacific Ocean (ESP, light green). Thin curves are the annual mean anomalies, while thick curves indicate nine-year running means. (**b**) Composite difference of SST and 10 m wind velocity between positive and negative IPO phases. (**c**) Same as (**b**) but for isopycnal temperature between 25 and 26 kg m^−3^. GrADS version 1.9b4 (Free Software - http://cola.gmu.edu/grads/gadoc/COPYRIGHT) was used for this figure.

One remaining question is the origin of the negative temperature anomalies in the subsurface South Pacific Ocean. To investigate their dominant time scale, we calculated time series of the nine-year running mean of isopycnal temperature anomalies averaged in the western off-equatorial South Pacific Ocean (WSP; 180°E–160°W and 15°S–8°S; the left box shown in Fig. 4c) from 1947 to 2014. The WSP index indicated that the negative anomalies have been persistent since 2000 (magenta curve of Fig. 4a). Interestingly, the WSP index reveals a dominant decadal variability. We also calculated the Interdecadal Pacific Oscillation (IPO)^12^ index derived from an Empirical Orthogonal Function (EOF) analysis of the nine-year running mean of SST anomalies in 100°–70°W and 30°S–40°N; we found that the WSP index fluctuated in harmony with the IPO index. These indices are similar to other IPO indices calculated by several previous studies^10^. The composite difference map of SST based on the IPO index (positive minus negative phases) showed a decadal El Niño-like pattern, which is a typical pattern of IPO (Fig. 4b). The map of the composite isopycnal temperature indicated a warmer (colder) condition in the western (eastern) off-equatorial South Pacific Ocean during a positive phase of IPO (Fig. 4c). Several previous studies have reported that the subsurface ocean temperature signals in the South Pacific drive the tropical SST changes associated with the climate shift in the mid-1970s when the IPO turned from the negative to positive phase^11^,^13^,^14^. A recent numerical study showed that the tropical bidecadal variability of SST originates via isopycnal advection of temperature anomalies from the eastern off-equatorial South Pacific Ocean (ESP) to the tropics, acting as delayed negative feedback^15^. We also defined the isopycnal temperature in the ESP region (120°W–90°W and 20°S–13°S, the right box shown in Fig. 4c). Time series of the indices in Fig. 4a show that the ESP index preceded the IPO and the WSP index. During an IPO positive phase, southward wind anomalies along the west coast of South America cause negative temperature anomalies in the isopycnal surface in the ESP region through the diapycnal mixing process^15^. The negative anomalies in the ESP region are advected to the western boundary on a decadal timescale and then northward to the equator^15^. Once the anomalies reach the equatorial strip, they are advected eastward and exposed to the sea surface. These anomalies dampen the previous positive IPO phase and trigger the next IPO phase. In recent years, the isopycnal temperature anomaly in the ESP region changed from negative to positive (Fig. 4a), suggesting that the negative phase of IPO that has persisted since 2000 will end in the near future.

### Extended partial-blocking prediction

So far, we have focused on the specific ENSO event in 2014. Meanwhile, the negative temperature anomalies in the subsurface South Pacific Ocean have continued to exist since 2000 (Fig. 4a). To examine the impact of the subsurface decadal temperature anomalies in the off-equatorial South Pacific Ocean on other ENSO events, we extended the partial-blocking hindcast (initialized on 1 November of each year) back to 1990. The differences between the original hindcasts and the partial-blocking hindcasts after 2004 (the period of negative isopycnal anomalies) were compared to those in the 1990s (the period of positive isopycnal anomalies). The difference in composite SST patterns (the original hindcast minus the partial-blocking hindcast) indicated that after 2004, the negative isopycnal temperature anomaly in the South Pacific Ocean encouraged a cooling tendency in the tropical Pacific SST eight to ten months after the prediction began (Fig. S3b). Nino3.4 indices from each experiment (Fig. S3a) indicated that the impact was also clear in the weak El Niño events during 2004–2005. Given that individual ENSO events are also affected by factors such as WWEs, it is not surprising that the cooling effect of negative anomalies in the South Pacific Ocean was not always robust. Interestingly, in the 1990s, when the positive isopycnal temperature anomaly existed in the South Pacific Ocean, the pattern was asymmetrical to that of the 2000s; expected SST warming was only visible in the south off-equatorial region, and the equatorial SST was cooled in the central to eastern Pacific. One possible reason is the large variance in the year-to-year isopycnal temperature anomalies in the 1990s. The WSP index sometimes had a negative value in the 1990s, although the low-frequency anomaly was positive (Fig. 4a). On the other hand, the interannual isopycnal temperature variability was weak after 2000; as such, a positive anomaly rarely happened. Although the reason for the difference between the two eras has yet to be explained, the above results indicate that the negative subsurface temperature anomalies in the South Pacific during the last decade contributed to the suppression of recent El Niño events.

## Discussion

With respect to the El Niño of 2014, some climate scientists pointed out that the absence of sustained westerly wind anomalies from late spring to early summer was the main cause of the unmaterialized El Niño^1^,^2^,^4^ and the basinwide warm anomalies in the north of the equator in 2014^16^. Our study showed that the subsurface negative anomalies in the South Pacific Ocean impacted the equatorial surface process and contributed to the formation of cold anomalies over the cold tongue region. The colder SST in the eastern tropical Pacific was also favourable to the suppression of WWEs in 2014. Thus, our finding might provide insight to understanding why the winds and the ocean failed to fully engage with each other.

A few previous studies raised the possibility that the moderate El Niños in the recent decades are attributed to the background slow changes associated with global warming^17^. Our study showed the new possibility of a role of the background slow cooling in the South Pacific subsurface ocean since early 2000s due to the Pacific decadal variability.

Even with sophisticated climate models for seasonal forecasting, predicting irregularities of the ENSO cycle is still challenging. In 2015, an El Niño has been developing since the summer, and most state-of-the-art forecasting models predict the development of a large El Niño in the following winter. In the isopycnal surface of the off-equatorial South Pacific Ocean, temperature anomalies are still negative, but their amplitude is weaker compared to 2014, suggesting that the condition of 2015 is more favourable for the development of El Niño than that of 2014. By monitoring the patterns of isopycnal temperature in the South Pacific Ocean, we can know in advance whether an ENSO event will easily progress or not. From this perspective, we can re-acknowledge the importance of continuous global *in-situ* ocean observations like the Argo profiling float.

## Methods

### The observational data set

The observed temperatures were obtained from the ProjD dataset (http://rda.ucar.edu/datasets/ds285.3/)^18^,^19^, the gridded monthly objective analysis with the latest observational databases [the World Ocean Database (WOD05), World Ocean Atlas (WOA05), and Global Temperature-Salinity Profile Program (GTSPP) provided by the National Oceanographic Data Center of USA (NODC)], and an SST analysis [Centennial *in-situ* Observation Based Estimates of variability of SST and marine meteorological variables (COBE SST)^20^,^21^]. Biases from the expendable bathythermographs (XBTs) and mechanical bathythermographs (MBTs) were eliminated in the objective analysis. The data were linearly interpolated to each day and to the ocean model grid. The observed anomaly was defined with a reference period between 1979 and 2008. For the 10 m wind velocity, we used the 55-year Japanese Reanalysis (JRA55) dataset from January 1958 onwards^22^.

The South Pacific cold anomaly in the 2000s is a point of focus in this study. We confirmed that this feature was also robust in other datasets such as the National Centers for Environmental Prediction (NCEP) Global Ocean Data Assimilation System (GODAS)^23^.

### MIROC5 seasonal prediction

To perform our seasonal hindcasts, we used a state-of-the-art AOGCM called MIROC5^6^. The atmospheric part of MIROC5 has a resolution of horizontal triangular spectral truncation at total wave number 85 (T85) and 40 vertical levels with the model top around 3hPa. For the ocean part, the zonal resolution is 1.4°, and the meridional resolution ranges from 0.5° (near the equator) to 1.4° (higher latitudes). There are 49 vertical levels spanning 2.5 m in the top level and 250 m in the bottom level. In the initialization process^24^, the observed temperature and salinity anomalies in the ocean were incorporated into model anomalies under the 20th century and RCP4.5 climate forcing. The observed temperature and salinity data were obtained from the ProjD dataset^18^,^19^.

In the forecasting system^5^, initial states were taken from the above ocean data assimilation system, while atmospheric variables were initiated from the National Centers for Environmental Prediction (NCEP) reanalysis^25^. An eight-member ensemble was available that was formed by the Lagged Average Forecast (LAF^26^) method with 12-hour intervals. In the analyses, anomalies were calculated as deviations from monthly climatology from 1979 to 2008, where mean biases for each lead month and each ensemble member estimated from hindcasts were also removed. One-year hindcasts were performed initialized on 1 Februaly, 1 May, and 1 August, and 1 November of each year. ENSO prediction skill of MIROC5 model was fully examined in our previous paper^5^. We confirmed that the skill of MIROC5 is comparable to the other state-of-the-art seasonal forecasting models. In this study, we used one-year predictions initialized on 1 November of each year. All eight members initialized on 1 November 2013 were used for analysing El Niño 2014, and five members of LAF with 24-hour intervals were used for the extended analysis from 1990 to 2013.

### Partial-blocking prediction

To eliminate the influence of anomalies from the off-equatorial South Pacific Ocean, model temperature and salinity within a water column in the South Pacific around 10°S (8°–12°S, 125°E–70°W) were restored to the monthly climatological values with a restoring time scale of 10 days. Here, temperature and salinity above a depth of 50 m were not restored in order to avoid reduction in surface atmospheric variability due to SST restoring. Such a partial-blocking method was also used in a previous study to examine South Pacific origin of the IPO^12^. Other conditions of the partial-blocking prediction were the same as the original prediction. A five-member ensemble of LAF initialized on 1 November of each year was available from 1990 to 2013.

### Tracer experiment

We conducted a tracer experiment to track the path of temperature anomalies under the same conditions of the original hindcast initialized on 1 November 2013. A passive tracer with concentration of 1.0 was prescribed in the pycnocline in the western off-equatorial South Pacific Ocean (see Fig. 3a) at the initial condition. The tracer was transported by ocean velocities without diffusion processes or surface fluxes.

## Additional Information

**How to cite this article**: Imada, Y. *et al*. South Pacific influence on the termination of El Niño in 2014. *Sci. Rep*. **6**, 30341; doi: 10.1038/srep30341 (2016).

## Supplementary Material

Supplementary Information
