# Supplementary material for: South Pacific influence on the termination of El Niño in 2014
Source: Sci Rep. 2016 Jul 28;6:30341. doi: 10.1038/srep30341 (PMC4964616; doi:10.1038/srep30341)
Supplement: Supplementary Information [file srep30341-s1.pdf]

Supplementary Materials for

South Pacific influence on the termination of El Niño in 2014

Y. Imada, H. Tatebe, M. Watanabe, M. Ishii, M. Kimoto

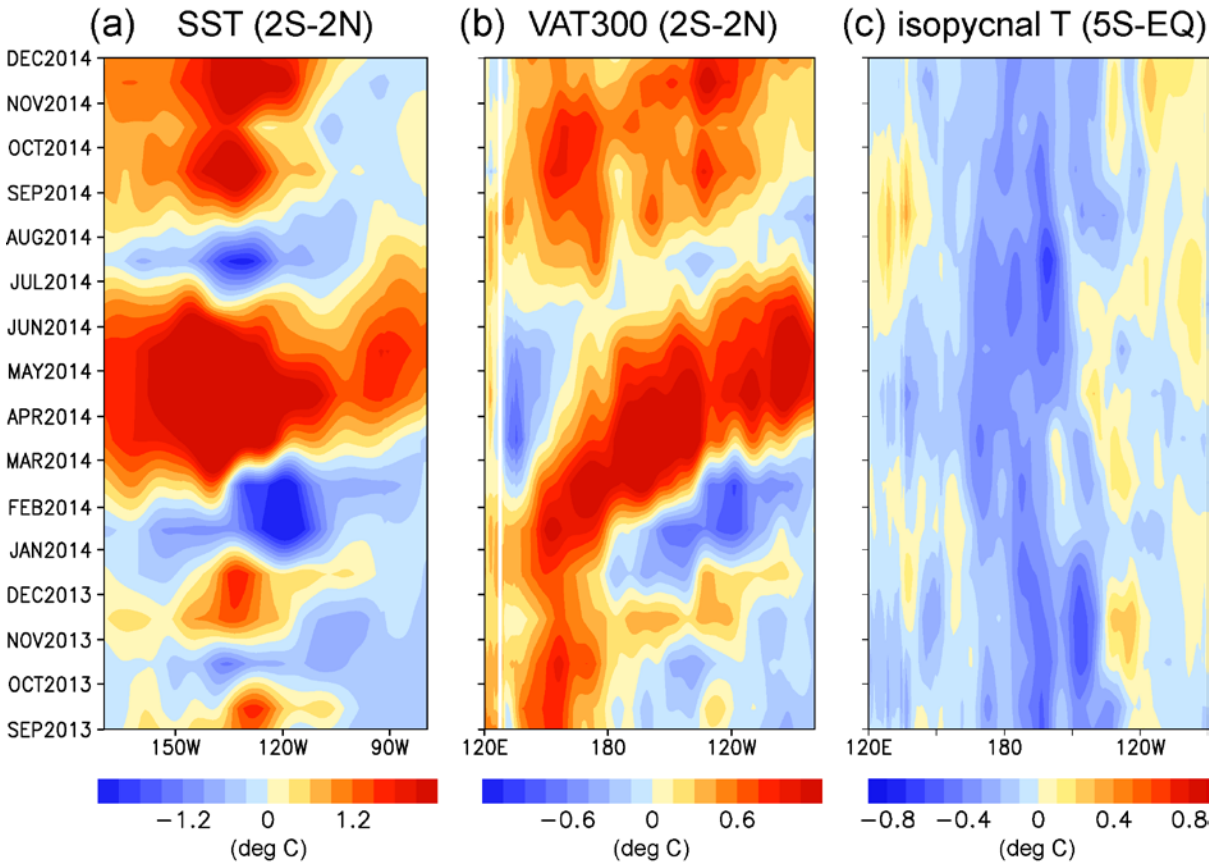

Figure S1 | Observed anomaly distribution near the equator. **a**, **b**, and **c**, Time-longitude plots showing observed anomalies of SST (**a**), vertical averaged temperature from the surface to a depth of 300 m (**b**), and ocean temperature at the isopycnal layer between 25.2 and 26.6 kg/m^3 (**c**).

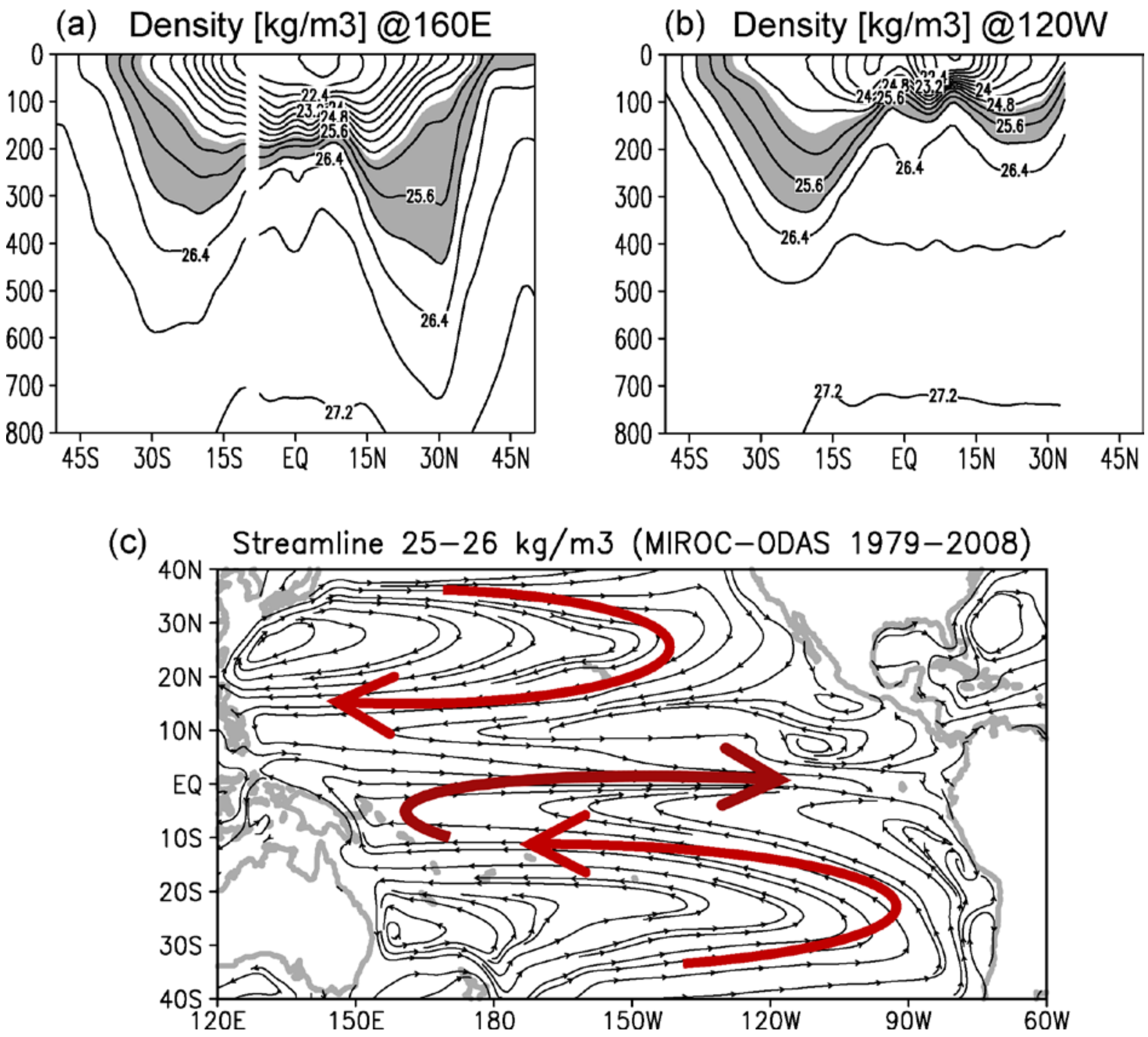

Figure S2 | Reference information for the isopycnal layer. a and **b**, Longitude-depth cross-sections of observed mean density at 160°E (**a**) and 120°W (**b**). The shading indicates the isopycnal layer between 25 and 26 kg/m^3. **c**, Mean stream lines (averaged from 1979 to 2008) in the isopycnal layer between 25 and 26 kg/m^3 from the MIROC5 ocean data assimilation. GrADS version 1.9b4 (Free Software - http://cola.gmu.edu/grads/gadoc/COPYRIGHT) was used for this figure.

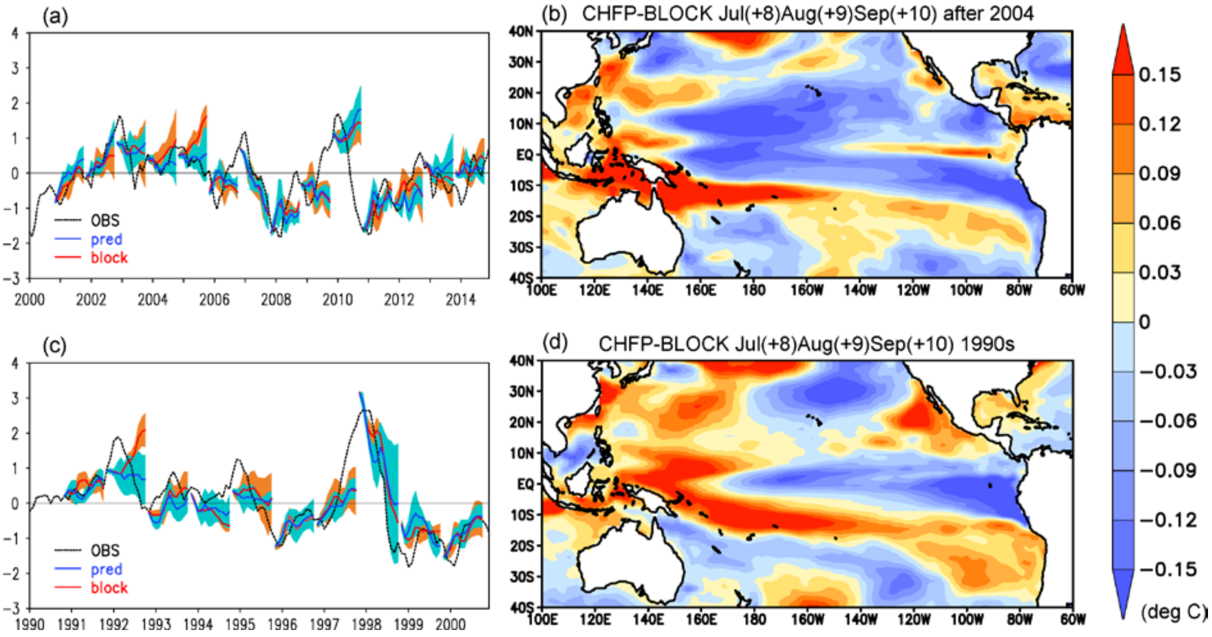

Figure S3 | Results of the extended hindcasts and partial-blocking predictions. a and **c**, Nino3.4 indices after 2000 (**a**) and in the 1990s (**c**) for the observation (black line), the MIROC5 prediction (blue line with light blue shading), and the partial-blocking prediction (red line with orange shading). Shading shows the range of the ensemble spread. **b** and **d,** Composite maps of SST differences between the MIROC5 hindcast and the partial-blocking prediction, averaged from the eighth to the tenth lead times (initialized on 1 November each year) for 1990–1999 (**b**) and 2004–2013 (**d**). GrADS version 1.9b4 (Free Software - http://cola.gmu.edu/grads/gadoc/COPYRIGHT) was used for this figure.
